# Role of NRP1/HDAC4/CREB/RIPK1 Axis in SARS‐CoV2 S1 Spike Subunit‐Induced Neuronal Toxicity

**DOI:** 10.1096/fba.2025-00005

**Published:** 2025-05-30

**Authors:** Luca Sanguigno, Natascia Guida, Mariarosaria Cammarota, Silvia Ruggiero, Angelo Serani, Francesca Galasso, Vincenzo Pizzorusso, Francesca Boscia, Luigi Formisano

**Affiliations:** ^1^ Division of Pharmacology, Department of Neuroscience, Reproductive and Dentistry Sciences, School of Medicine Federico II University of Naples Naples Italy; ^2^ Department of Neuroscience and Brain Technologies Istituto Italiano di Tecnologia Genova Italy

**Keywords:** cAMP response element‐binding protein (CREB), COVID‐19, histone deacetylases (HDACs), oxygen glucose deprivation/reoxygenation (OGD/Rx), receptor‐interacting serine/threonine‐protein kinase 1 (RIPK1), Spike

## Abstract

Severe acute respiratory syndrome coronavirus 2 (SARS‐CoV2) is associated with neurological symptoms, but the molecular mechanisms have not yet been identified. Since the S1 subunit (S1) of the envelope of the SARS‐CoV2 Spike glycoprotein can reach the CNS, we studied whether S1 could cause neuronal death in a direct manner. Transfection of the S1 plasmid in SH‐SY5Y cells reduces cell survival in a time‐dependent manner, whereas the overexpression of the S2 subunit does not. Notably, isoform 4 of histone deacetylases (HDAC4) is involved in S1‐induced cell toxicity, whereas, among the different cell death drug inhibitors, only the necroptosis blocker Necrostatin‐1 counteracted the neurodetrimental effect of S1. Coherently, an increase of the necroptosis marker receptor‐interacting serine/threonine‐protein kinase 1 (RIPK1) and a reduction of its transcriptional repressor cAMP response element‐binding protein (CREB) occur in S1‐overexpressing cells. Noteworthy, HDAC4 interacts with CREB determining its protein reduction and the consequent increase of RIPK1. Importantly, we found that S1 recombinant protein (S1rp), through the internalization of the surface receptor Neuropilin 1 (NRP1), but not via Angiotensin‐Converting Enzyme 2 (ACE 2) receptor, enters the cytoplasm causing cell death in differentiated SH‐SY5Y cells. Finally, in accordance with other papers demonstrating that COVID‐19 patients had more severe ischemic strokes with worse outcomes, we found that S1rp increased oxygen glucose deprivation/reoxygenation‐induced toxicity in an additive manner, via the NRP1/HDAC4/CREB/RIPK1 pathway. In conclusion, this is the first report identifying the molecular determinants involved in Spike S1‐induced neurotoxicity.

## Graphical abstract

Extracellular SARS‐CoV2 Spike‐S1 protein via NRP1 internalizes in the cytosol of SH‐SY5Y cells. Once inside, it upregulates the epigenetic eraser HDAC4, which binds transcriptional factor CREB, causes RIPK1 up‐regulation, and therefore necroptotic cell death.

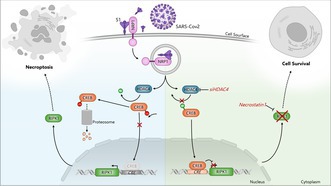

**Abbreviations**
ACE2: angiotensin‐converting enzyme 2
APP: amyloid precursor protein
BBB: blood–brain barrier
ChIp: chromatin immunoprecipitation
CNS: central nervous system
CREB: cAMP response element‐binding protein
HDAC: histone deacetylase
IL‐6: interleukin‐6
MLKL: mixed lineage kinase domain like pseudokinase
Nec‐1: Necrostatin‐1
NRP1: Neuropilin 1
OGD/Rx: oxygen and glucose deprivation/reoxygenation
RIPK1: receptor‐interacting serine/threonine‐protein kinase 1
RIPK3: receptor‐interacting serine/threonine‐protein kinase 3
S1rp: S1 recombinant protein
SARS‐CoV2: severe acute respiratory syndrome coronavirus 2
SIRT: sirtuin
TNF‐α: tumor necrosis factor‐α

## 1 Introduction

Several pieces of evidence demonstrated that COVID‐19 patients not only showed respiratory‐related symptoms, but also manifested mild and severe neurological disorders [1]. The neurological complications of COVID‐19 include large vessel occlusion, ischemic stroke, intracranial hemorrhage, encephalitis, myelitis, Guillain‐Barre syndrome, status epilepticus, posterior reversible encephalopathy syndrome, and hypoxic–ischemic encephalopathy [2, 3].

Severe acute respiratory syndrome coronavirus 2 (SARS‐CoV2) could enter the brain through the: (1) nasal cavity, which could bring the virus to the olfactory bulb via the olfactory nerves; (2) eyes, which could lead the virus to the occipital cortex via the optic nerve; (3) respiratory tracts, which can allow the virus to invade the blood system and consequently cross the blood–brain barrier (BBB) via transcellular, paracellular pathways or by intracellular cargo proteins [1]. Notably, SARS‐CoV2 infection in brain organoids and human cerebral tissues can cause neuronal death [4] but, since no detectable viral SARS‐CoV2 RNA was found in cerebrospinal fluid (CSF), it has been proposed that viral infection in the brain is not the main reason for cell death.

An alternative explanation of the central nervous system (CNS) toxic effect of SARS‐CoV2 may be due to direct effects of the structural proteins of the virus, such as the Spike glycoprotein that has been found in cortical neurons of patients who died of COVID‐19 [5]. The Spike protein is essential for the virus attachment and entrance to neuronal cells, which is promoted by the angiotensin‐converting enzyme 2 (ACE2) [6] or neuropilin‐1 (NRP1) [7] receptors present on the neuronal cell surface. Once inside the cells, Spike is cleaved into S1 and S2 subunits, a mechanism that is responsible for the virus internalization and that facilitates the viral‐cellular fusion machinery [8]. This process causes S1 subunit dissociation from the virus and the consequent release from the cells; indeed, S1 protein has been found in blood plasma, serum [9] and urine of COVID‐19 patients [10]. It has been found that intravenous (IV) injection or intranasal administration of S1, by crossing the blood‐brain barrier, determines its ingress to the brain parenchyma [11] and causes endothelial damage by activating caspase3, interleukin‐6 (IL‐6), and tumor necrosis factor‐α (TNF‐α). On the contrary, the S2 Spike subunit, similarly IV administered, was not found in the brain [12].

Notably, primary human cortical neurons incubated with S1 Spike subunit protein induce aberrant neuritic varicosities [13], which is a sign of neuronal damage in several neurological diseases [14]. Recently, Ali et al. demonstrated that skull marrow microinjections of S1 Spike in the mouse brain increase the expression levels of cleaved caspase‐3 and the amyloid precursor protein (APP) [15].

On the other hand, the epigenetic erasers histone deacetylases (HDACs) have been found to be involved in the pathophysiology of neurological mechanisms occurring during COVID‐19 infection. Indeed, HDACs contribute to the entrance of the virus into the CNS and facilitate its replication by up‐regulating the expression of the viral receptors ACE2 and NRP1 on the cell membrane surface [16, 17]. Four different classes of HDACs are known: class I HDACs (1–3 and 8) are constitutively nuclear proteins; class II HDACs (4–7, 9, 10, and) are expressed in a cell‐specific manner and shuttle between the nucleus and cytoplasm; class III HDACs or sirtuins consist of seven members (SIRT1–SIRT7); and class IV HDACs, currently composed of one member, HDAC11, of which little is known [18].

Notably, we found that HDAC4 knocking down is neuroprotective when cortical neurons are exposed to an in vitro model of stroke or to environmental neurotoxicants [19, 20, 21]. HDAC4‐dependent molecular mechanisms of neuronal death could be different, for example: (1) excitotoxic glutamate, by increasing HDAC4, activates caspase 3 and induces neuronal apoptosis [22], whereas (2) in a murine model of Duchenne muscular dystrophy, HDAC4 increasing and activation of receptor‐interacting serine/threonine‐protein kinase 1 (RIPK1) determines necroptotic cell death [23]. Necroptosis is a an alternative programmed cell death triggered by RIPK1 that is activated once caspase‐8 is reduced and RIPK3 and MLKL are increased [24]. Notably, elevated plasma concentrations of the necroptosis‐related proteins RIPK1, RIPK3, and Mixed lineage kinase domain like pseudokinase (MLKL) have been found in patients with moderate and severe COVID‐19 disease [25]. At the transcriptional level, RIPK1 is down‐regulated by its transcriptional repressor cAMP response element‐binding protein (CREB); indeed, its reduction after polychlorinated biphenyl (PCB)‐95 exposure up‐regulates RIPK1 mRNA, thus determining neuronal cell death [26]. Furthermore, it has been demonstrated that the replication of SARS‐CoV2 RNA was inhibited after knockdown of CREB with small interfering RNA [27].

Since we demonstrated that HDACs, CREB, and RIPK1 could be connected in several neurotoxic pathways [19, 20, 21, 28, 29], and are individually related to COVID‐19 physiopathology [25, 27, 30], herein, we investigated the possible relationship between Spike S1‐neurotoxicity and HDACs, CREB, and RIPK1 expression in SH‐SY5Y cells transiently transfected with the S1 vector or exposed to the S1 protein.

Moreover, since COVID‐19 associated ischemic strokes are more severe with worse functional outcomes and higher mortality than non‐COVID‐19 ischemic strokes [31], we studied, by using an in vitro model of brain ischemia, whether S1 could increase the severity of brain injury by modulating a specific HDACs isoform and RIPK1.

## 2 Materials and Methods

### 2.1 Reagents, Drugs and Antibodies

All reagents were supplied by Sigma (Milan, IT). Oligonucleotides were synthesized by Eurofins Genomics (Ebersberg, Germany). The following siRNAs were used for human proteins: HDAC1, HDAC2, HDAC3, HDAC4, HDAC5, HDAC7, and HDAC9 [32, 33], NRP1 (sc‐36038) and ACE2 (sc‐41400). The following siRNAs were used for rat proteins: NRP1 (orb1830264), ACE2 (orb1829102), HDAC4 [21], RIPK1 [26], and CREB purchased from Dharmacon. For all experiments, the siRNA CONTROL (siCTL) was from QIAGEN (All Stars Negative Control siRNA, cod: 1027280). The SARS‐CoV2 expression plasmid constructs were: (1) pcDNA3.1 + SARS‐Spike‐C9, (Addgene Cod: 145031) for Spike Vector, (2) pCMV3‐S1, (Sino Biological, Cod: VG40591‐UT) for S1 subunit vector, and (3) pCMV3‐S2, (Sino Biological, Cod: VG40590‐UT) for S2 subunit vector. The plasmid used for CREB overexpression was gently gifted by Marc Montminy (Plasmid #22394). For all experiments with constructs transfection, pcDNA3.1 has been used as the empty vector (EV). Necrostatin‐1 (Nec‐1;10 μM), z‐VAD‐fmk (ZVAD; 50 μM), Calpeptin (Calp; 30 μM), MG132 (10 μM) [20, 26, 33] and Retinoic Acid (RA; 10 μM) [34] were prepared and used as previously reported. Sodium Azide (SIGMA, Cod: S2002, 30 mM). Culture media and sera were purchased from Invitrogen. All chemicals were diluted in cell culture medium. 0.1% dimethyl sulfoxide (DMSO) was the final concentration used as the vehicle that did not cause cellular toxicity. SARS‐CoV2 Spike S1 recombinant protein (S1rp) was purchased from GeneScript (Cod. Z03501‐100; stock solution 2.01 mg/mL). The antibodies used were the following: Anti‐C9 tag (MyBioSource, MBS430088); Anti‐SARS‐CoV‐2 Spike Protein (Invitrogen, PA5‐114451); Anti‐Spike S2 (GeneTex, 135386); Anti‐Spike S1 (Invitrogen, PA5‐114451); Anti‐NRP1 (GeneTex, GXGTX127947); Anti‐ACE2 (SantaCruz, sc‐390851); Anti‐CREB (Cell Signaling, 9194S). Anti‐HDAC4 [21]; Anti‐RIPK1; Anti‐CREB; and Anti‐Actin [26], which have been used in previous studies.

### 2.2 Cell Cultures

Human neuroblastoma SH‐SY5Y cells were obtained and maintained as already reported [26, 33]. To induce differentiation, retinoic acid (RA) was added 24 h after plating at a final concentration of 10 μM in culture medium for 5 days. The cellular density for: (1) MTT, LDH, and Luciferase assays was 1 × 10^6^ cells/well in 24 well plates; (2) qRT‐PCR was 5 × 10^6^ cells/plate in 60 mm plates; (3) Western blot and Immunoprecipitation was 15 × 10^6^ cells/plate in 100 mm plates.

### 2.3 Transfection of SH‐SY5Y Cells

SH‐SY5Y cells were transfected with Lipofectamine 2000 (Thermo Fisher, cod: 11668027), following the manufacturer's instructions. Twenty‐four hours after plating, SH‐SY5Y cells (50% confluence) were singly transfected with the following constructs: (1) pcDNA3.1, (2) Spike Vector, (3) S1 Vector, and (4) S2 Vector. The transfection was blocked after 24, 48, and 72 h for time course experiments. Protein expression generated from: (1) Spike Vector transfection was detected with Anti‐C9 tag; (2) S1 Vector transfection was detected with Anti‐Spike S1; and (3) S1 Vector transfection was detected with Anti‐Spike S2. The siRNAs against specific HDACs (50 nM) and the plasmid overexpressing CREB were co‐transfected with S1 Vector for 48 or 72 h. The day after S1 Vector transfection, cells were treated with vehicle, Nec‐1, ZVAD, Calpeptin, and MG132 for 3 h, diluted in fresh medium (1% FBS); the experiments were blocked 24 h after drug treatment. Transfection efficiency was almost 50%.

### 2.4 Spike Protein Treatment in Differentiated SH‐SY5Y Cells

S1rp (Stock solution 2 mg/mL PBS) was dissolved in the culture medium of differentiated SH‐SY5Y cells to reach different concentrations (0.025–0.1 μg/mL) for dose–response experiments. The culture medium was changed and fresh S1rp was added daily. CTLs were pretreated with vehicle (PBS) siRNA transfection (50 nM) was performed with Lipofectamine 2000 after 5 days RA differentiation in SH‐SY5Y cells [33]. Twenty‐four hours after siRNA transfection, cells were treated with S1rp 100 ng/mL for 72 h.

### 2.5 Combined Oxygen and Glucose Deprivation and Reoxygenation

OGD was performed in SH‐SY5Y as already reported [33]. Cells were incubated in a medium containing: 116 mM NaCl, 5.4 mM KCl, 0.8 mM MgSO_4_, 26.2 mM NaHCO_3_, 1 mM NaH_2_PO_4_, 1.8 mM CaCl_2_, 0.01 mM glycine, and 0.001% w/v phenol red, previously saturated with 95% N_2_ and 5% CO_2_ at 37°C for 20 min. Afterward, cells were placed in a hypoxic chamber for 4 h under the following conditions: temperature 37°C, atmosphere 5% CO_2_, and 95% N_2_. To conclude OGD, cells were removed from the hypoxic chamber and placed in a normal medium for 72 h of reoxygenation (Rx). To study the effect of S1rp on OGD/Rx‐induced cell death, SH‐SY5Y cells were treated with S1rp (1 μg/mL) for 72 h during the Rx phase. siRNA transfection (50 nM) was performed 24 h before the OGD/Rx insult.

### 2.6 Western Blot Analysis, Immunoprecipitation, and Cell Fractionation

Cells were washed and collected in cold Phosphate Buffered Saline (PBS; Sigma, Milan IT). The cell pellet was resuspended in a RIPA lysis buffer (sc‐24948, Santa Cruz Biotechnology) with 1× protease inhibitor and incubated on ice for 2 h. The lysate was centrifuged at 14,000 rpm for 20 min at 4°C to obtain total proteins. Immunoprecipitation was performed as previously reported [20]. Specifically, 1.5 mg of protein was immunoprecipitated overnight at 4°C using 2 μg of the following antibodies: Anti‐CREB mouse monoclonal (cod. sc‐271, Santa Cruz Biotechnology) and Normal Mouse IgG‐AC (cod: sc‐2343, Santa Cruz Biotechnology) as a negative control. The immune complexes were then precipitated through the use of 50 μL of Protein A/G PLUS‐Agarose beads (sc‐2003, Santa Cruz Biotechnology). The immunoprecipitates were then subjected to Western blot analysis for HDAC4 antibody.

Isolation of cytosolic and membrane fraction was performed as previously described [35]. Specifically, cells were collected in a 15‐mL falcon tube and centrifuged at 1000 g for 5 min. The pellet was resuspended in 300 μL of buffer solution (Buffer A), which is composed of: 50 mM TRIS pH 8.0; 0.5 mM DDT, 0.1% NP‐40; protease and phosphatase inhibitors, and homogenized with a hand microtube homogenizer. Next, the lysate was transferred to a 1 mL syringe and passed through a 26‐gauge needle (10 times) and disrupted. The suspension was centrifuged at 1000 g for 10 min. After this centrifugation, the pellet that contains membrane fraction (from endosomes, Golgi, plasma membrane, endoplasmic reticulum and secretory vesicles) was suspended in NP‐40 free Buffer A, stood on ice for 10 min, and re‐centrifuged at 1000 g for 10 min. The precipitate obtained was suspended again in Buffer A containing 1% (v/v) NP‐40, stood on ice for 60 min, and further centrifuged at 16,000 g for 20 min. The supernatant obtained was the cytosol fraction. All the procedure was performed in a cold room to limit the action of proteases and phospholipase. The purity of the preparation was assessed by evaluating the expression of Tubulin and NCX1 as cytosolic and membrane markers [36], respectively. All protein extracts were quantified by Bradford Protein Assay (Bradford Reagent, Biorad, #5000006) and separated on polyacrylamide gels (Precast Protein Gels 4%–20%, Biorad, #4561096). Proteins were transferred onto nitrocellulose membranes (Nitrocellulose Transfer Packs, Biorad, #1704158) using the Semi‐Dry TransBlot System. Primary and secondary antibodies used are reported in the Reagents, Drugs and Antibodies paragraph. Analysis and quantification have been performed as already reported [33].

### 2.7 qRT‐PCR

Total RNA extraction from cells was performed using TRI Reagent (Sigma, cod: T9424), according to the vendor's directions. Retrotranscription was performed on 2 μg of RNA, using the reagents contained in the Applied Biosystems High‐Capacity cDNA Reverse Transcription Kit (Thermo Fisher Scientific, cod: 4368814) following the manufacturer's directions and protocol (10 min at 25°C, 2 h at 42°C, 5 min at 85°C, ∞ 4°C). The obtained cDNA samples were amplified by RT‐qPCR with SYBR Green Real‐Time PCR Master Mix (Thermo Fisher, cod: 4309155) in triplicate with a protocol previously described [26]. PCR data were collected and analyzed by using ABI Prism 7000 SDS software (Applied Biosystems). The expression of the genes of our interest was normalized to the expression of the house‐keeping gene encoding for Ribosomal Protein L19 for human genes and HPRT for rat genes [26]. Changes in mRNA levels were shown as the average of relative quantification (RQ) values, obtained as the threshold cycle difference (Δ*C*_*t*_) between the target gene and the reference gene ($2^{-\Delta C_{t}}=\mathrm{RQ}$) [33]. The primers used in this paper were the following: ACE2 human FW: 5′‐AGA AAG CAG TCT GCC ATC CC‐3′; ACE2 human RV: 5′‐GCT GTC AGG AAG TCG TCC AT‐3′; NRP1 human FW: 5′‐TAG CTC CAA CGG GGA AGA CT‐3′; NRP1 human RV: 5′‐TAG CTC CAA CGG GGA AGA CT‐3′; RIPK1 human FW: 5′‐GGAGACTAGGTGGCAGGGTA‐3′; RIPK1 human RV: 5′‐TCTGCGATCTCGGCTTTCAG‐3′.

### 2.8 Transient Chromatin Immunoprecipitation Assay

For the transient chromatin immunoprecipitation (ChIP) assay, cells at 60% confluence were co‐transfected for 24 h with: (1) pGL3‐RIPK1‐promoter and (2) S1 Vector or EV. Antibodies used in the procedure include anti‐HDAC4, anti‐CREB, and polyclonal IgG as a negative control. Transfection conditions, ChIP assay procedures, and primer sequences have already been described by our group [26]. Amplification and quantification were performed as previously reported [26]. Results obtained from three different PCR experiments were normalized for the DNA input.

### 2.9 Luciferase Assay

The RIPK1 promoter (indicated as pGL3‐RIPK1) was cloned in the pGL3basic vector as previously published [26]. Specifically, to study the RIPK1 promoter activity, SH‐SY5Y cells were co‐transfected with 1.5 mg of total DNA vectors, including: (1) 800 ng of reporter constructs, that are pGL3‐basic or pGL3‐RIPK1; (2) 200 ng of the pRL‐TK vector; (3) 500 ng of S1 Vector. For siRNAs transfection, 50 nM of siCTL or siHDAC4 were co‐transfected with other plasmids. To overexpress CREB, 500 ng of CREB construct or empty vector were co‐transfected. For the MG132 experimental group, cells were treated 24 h after transfection with MG132 for 3 h. All experimental groups were analyzed 48 h after transfection with the Dual‐Luciferase Reporter Assay System Kit (Promega Italy, E1910). Luciferase activity was expressed as firefly‐to‐renilla ratio in arbitrary units.

### 2.10 MTT and LDH Assays

The 3‐[4,5‐dimethylthiazol‐2‐yl]‐2,5 diphenyl tetrazolium bromide (MTT) and lactate dehydrogenase (LDH) assays were performed as previously described in SH‐SY5Y [33]. For the MTT assay, culture medium was removed and cells were incubated in 500 μL of a 0.5 mg/mL MTT solution for 2 h at 37°C. Incubation was blocked by adding 500 μL of deacidified isopropanol to solubilize the formazan salt, and viability was read by measuring absorbance at 540 nm. The amount of LDH released into the extracellular medium was measured by using the kit from Cayman (cod: E‐BC‐K771‐M) DBA (Milan, Italy) following the manufacturer's instructions. For LDH experiments, 1% Triton X100‐treated cells were used as a positive control and its value was considered to be 100% cell death.

### 2.11 Confocal Immunofluorescence Analysis

Confocal immunofluorescence procedures in SH‐SY5Y cultures were performed as previously described [37, 38]. Cell cultures were fixed in 4% wt/vol paraformaldehyde in phosphate buffer for 30 min. After blocking with 3% BSA, cells were incubated with the primary antibodies for 24 h. The primary antibodies used were the following: rabbit polyclonal anti‐C9 Tag (1:1000, MyBioSource, MBS430088); SARS‐CoV2 Spike (1:500; #PA5‐114451, Invitrogen); rabbit polyclonal anti‐HDAC4 (1:200, sc‐11418; Santa Cruz Biotechnology); goat polyclonal anti‐RIPK1 (1:200, sc‐41169, Santa Cruz Biotechnology); mouse monoclonal anti‐CREB (1:400, 9194S, Cell Signaling); rabbit polyclonal anti‐Neuropilin 1 (1:400, GXGTX12794, GeneTex). Then, cells were incubated with corresponding fluorescence‐labeled secondary antibodies (Alexa488‐ or Alexa594‐conjugated anti‐mouse or anti‐rabbit IgG). Hoechst‐33342 (Merck, Millipore) was used to stain nuclei. Images were observed using a Zeiss LSM 700 laser (Carl Zeiss) scanning confocal microscope. Single images were taken with an optical thickness of 0.7 μm and a resolution of 1024 × 1024. All staining and morphological analyses were blindly conducted. Images were processed and analyzed with the public domain Java‐based image processing software ImageJ (National Institutes of Health, Bethesda, Maryland, USA). Quantification of HDAC4 and RIPK1 fluorescence was quantified in terms of pixel intensity by using the NIH image software, as described previously [38]. Briefly, digital images were taken with a 63× objective, and identical laser power settings and exposure times were applied to all the photographs from each experimental set. The number of cells displaying CREB immunoreactivity in the nucleus was determined by manual counting at ×40 magnification.

### 2.12 Statistical Analysis

Data were analyzed by Graph Pad Prism 5 software (Graph Pad Software Inc.). All bars in the figures represent the mean ± SD. Statistical differences between two experimental groups were analyzed with the Student's *t*‐test. Statistically significant differences between more than two experimental groups were evaluated by one‐way ANOVA, followed by Tukey's multiple comparison test.

## 3 Results

### 3.1 SARS‐CoV2 S1‐Spike but Not S2‐Spike Reduced Cell Survival in SH‐SY5Y Cells

In this study, we used SH‐SY5Y neuronal cells transiently transfected with the following vectors containing: (1) the genes for S1 and S2 subunits of the Spike protein of the SARS‐CoV2 (Spike Vector); (2) the SARS‐CoV2 Spike‐S1 gene (S1 Vector) and (3) the SARS‐CoV2 Spike‐S2 gene (S2 Vector). An empty vector (EV) was used as control.

**FIGURE 1:**
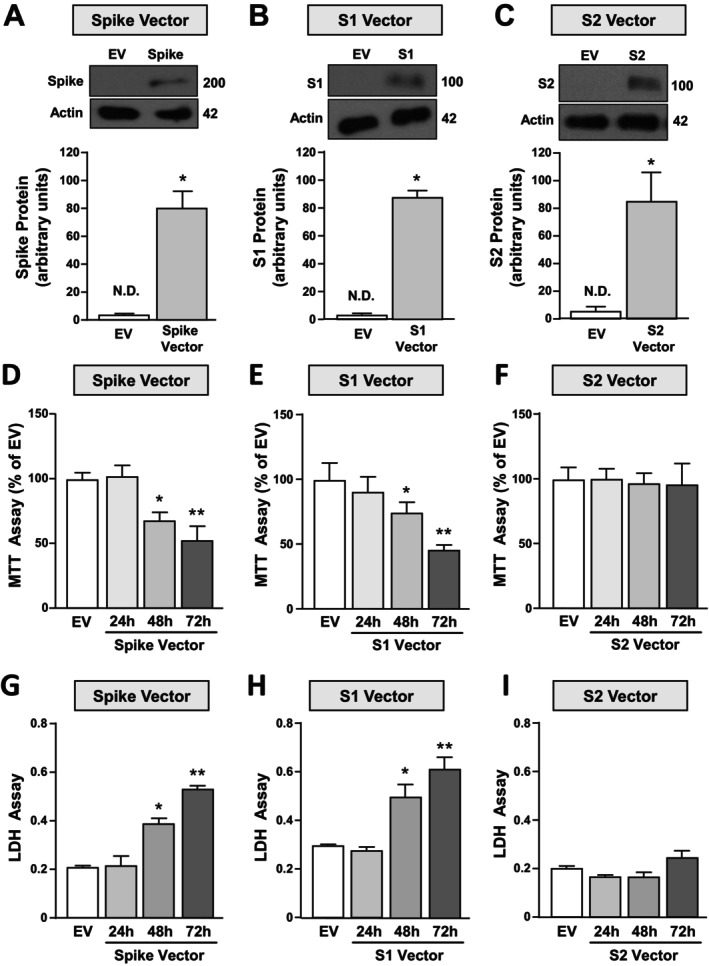
Effect of transfection of the following constructs: (1) empty vector, (2) Spike S1 + S2 Vector, (3) Spike S1 Vector and (4) Spike S2 Vector on Spike protein expression and cell viability in SH‐SY5Y cells. (A–C) Western blotting representative image and quantification of Spike protein in cells transfected for 48 h with the following vectors: Spike‐S1 + S2 (Spike Vector), Spike‐S1 (S1 Vector) and Spike‐S2 (S2 Vector) or with the empty vector (EV). (A) Spike was recognized by using an antibody against its C‐terminal C9 tag, whereas (B) Spike S1 and (C) Spike S2 subunits were detected by using two different specific antibodies (see Section 2). *p ≤ 0.05 versus EV by Student's *t* test (*n* = 4). (D–F) Cell viability measured by MTT assay and (G–I) cell death measured by LDH assay in SH‐SY5Y cells transfected with Spike Vector, S1 Vector and S2 Vector for 24, 48 or 72 h. *p ≤ 0.05 versus EV; **p ≤ 0.05 versus all by one‐way ANOVA analysis followed by Tukey's post hoc test (*n* = 4/5).

Spike, S1, and S2 Vectors‐transfected cells showed a strong expression in Spike, S1, and S2 protein expression, respectively, compared to EV‐transfected cells, thus confirming the transfection efficiency (Figure 1A–C). Importantly, cells transfected with Spike and S1 Vector, but not with S2 Vector, resulted in a time‐specific vitality reduction, as revealed by MTT (Figure 1D–F) and LDH assays (Figure 1G–I). These results indicate that 72 h is the time point inducing an approximately 50% reduction of cell vitality by Spike and S1 transfection (Figure 1D,E,G,H). Notably, Pileggi et al. also found an increase of LDH release in SH‐SY5Y cells stably transfected with S1 Vector. These experiments demonstrate that S1 is the subunit responsible for Spike neurotoxicity.

### 3.2 siRNA Against HDAC4, by Blocking RIPK1 mRNA and Protein Increase, Limited the S1‐Vector Induced Cell Death in SH‐SY5Y Cells

**FIGURE 2:**
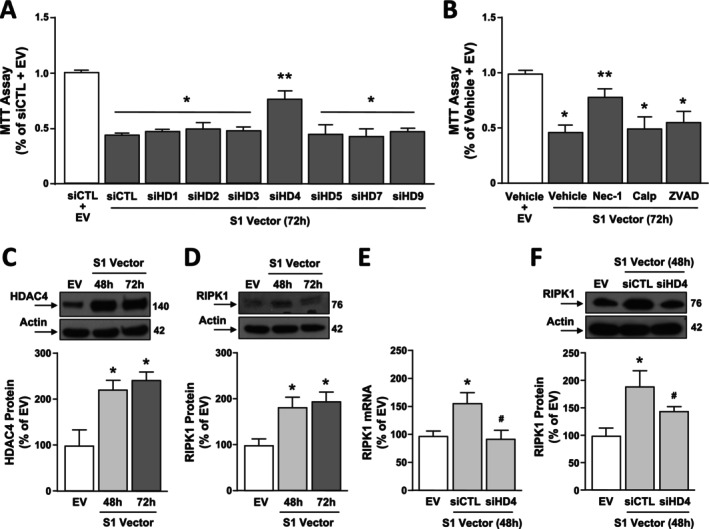
Effect of HDAC4 knockdown and Necrostatin‐1 to reduce S1 Vector‐induced (1) cell death and (2) RIPK1 mRNA and protein increase in SH‐SY5Y cells. (A) Cell vitality measured by MTT assay in SH‐SY5Y cells transfected with S1 Vector and treated with siRNA against HDAC1 (siHD1), HDAC2 (siHD2), HDAC3 (siHD3), HDAC4 (siHD4), HDAC5 (siHD5), HDAC7 (siHD7), HDAC9 (siHD9) or siRNA control (siCTL) for 72 h. (B) Cell vitality measured by MTT assay in SH‐SY5Y cells transfected with S1 Vector and treated with Vehicle, Nec‐1 (10 μM), Calpeptin (30 μM) or ZVAD (50 μM) for 72 h. *p ≤ 0.05 respect to control (siCTL + EV or Vehicle + EV); **p ≤ 0.05 versus all by one‐way ANOVA analysis followed by Tukey's post hoc test (*n* = 4/5). (C, D) Western blotting representative images and quantification of HDAC4 and RIPK1 proteins in SH‐SY5Y cells after 48 and 72 h transfection with S1 Vector. *p ≤ 0.05 versus control (EV) by one‐way ANOVA analysis followed by Tukey's post hoc test (*n* = 4). Effect of siHDAC4 on RIPK1 at (E) transcriptional and (F) protein level after 48 h of S1 Vector transfection. *p ≤ 0.05 versus control (EV); ^#^ *p* ≤ 0.05 versus siCTL + S1 Vector (48 h) by one‐way ANOVA analysis followed by Tukey's post hoc test (*n* = 3/4).

Since we previously demonstrated that HDACs knocking down is neuroprotective against several environmental neurotoxicants [20, 29, 32, 39], we evaluated the effect of siRNAs against HDACs (siHDACs) of class I (HDAC 1–3) and of class II (HDAC 4, 5, 7, and 9), enzymes that are basally expressed in these cells [29, 32], on Spike S1‐induced cell death. Notably, the efficiency of single siRNAs in reducing protein expression of the different HDACs has already been published. Interestingly, we found that only siHDAC4 determined a significant increase in cell viability in S1 Vector‐transfected cells (Figure 2A). Since it is known that HDAC4 up‐regulation could cause cell death by activating necrosis, apoptosis, or necroptosis [22, 23, 40] we used selective pharmacological inhibitors of these cell death pathways in order to identify which mechanisms occur following S1 vector transfection. It is noteworthy that only the necroptosis inhibitor Nec‐1, but not the necrosis and the apoptosis inhibitors Calpeptin and ZVAD, was able to partially prevent S1 vector‐induced cytotoxicity, as revealed by MTT assay (Figure 2B). Next, we investigated the correlation between HDAC4 and the main necroptosis mediator RIPK1. As shown in Figure 2C,D, 48 and 72 h of S1 Vector transfection increased HDAC4 and RIPK1 protein expression in SH‐SY5Y cells. More relevantly, siHDAC4 reverted at transcriptional and translational levels the RIPK1 up‐regulation induced by S1 subunit transfection (Figure 2E,F). These results indicate that S1 overexpression induced cell death via HDAC4 and RIPK1 increase.

### 3.3 siRNA Against HDAC4 or the Proteasomal Inhibitor MG132, by Restoring CREB Binding on RIPK1 Promoter Gene, Counteracts S1 Vector‐Dependent RIPK1 Up‐Regulation in SH‐SY5Y Cells

**FIGURE 3:**
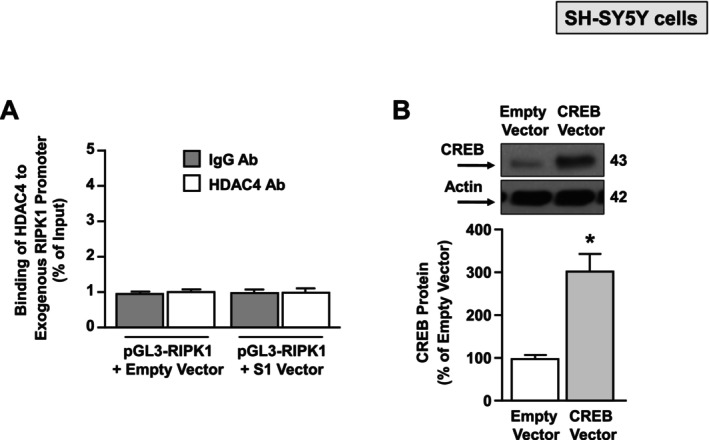
HDAC4 does not bind the RIPK1 human promoter in S1 Vector‐transfected SH‐SY5Y cells. (A) ChIP with HDAC4 and IgG antibodies followed by qPCR of the RIPK1 human promoter in SH‐SY5Y cells transfected for 72 h with: (1) pGL3‐RIPK1 + EV, (2) pGL3‐RIPK1 + S1 Vector (*n* = 3). The pGL3‐RIPK1 + EV group immunoprecipitated with IgG was defined as 1.0 and was used to compare the other experimental groups. (B) Western Blotting representative images with quantification of CREB protein levels in SH‐SY5Y cells transfected 48 h with a vector overexpressing CREB. *p ≤ 0.05 versus empty vector by unpaired Student's *t* test (*n* = 4).

**FIGURE 4:**
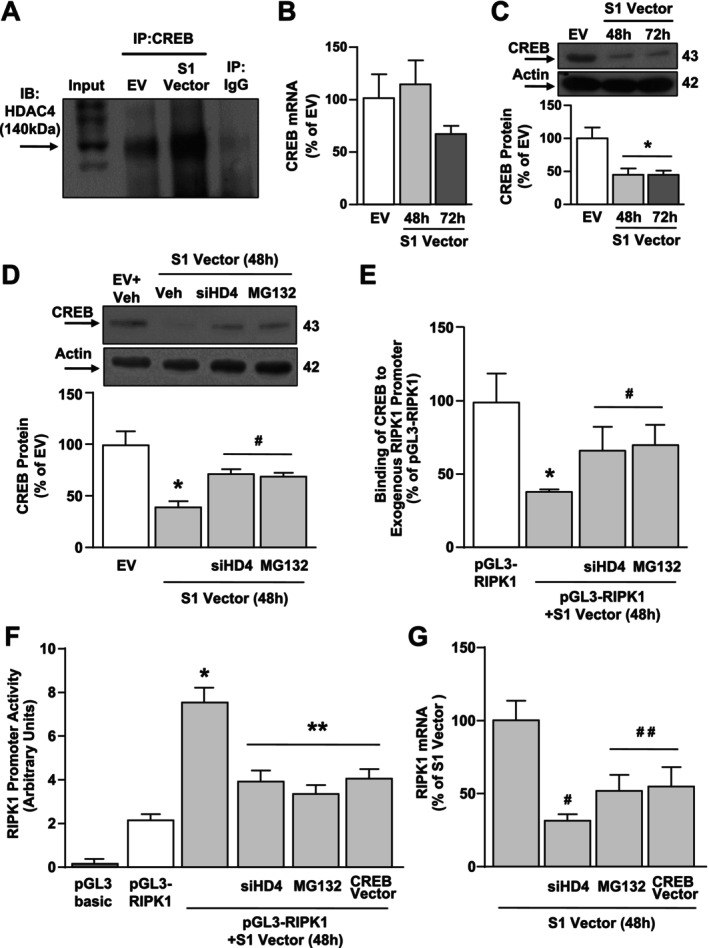
Effect of siHDAC4 and proteasomal inhibitor MG132 on S1 Vector‐induced down‐regulation of CREB in SH‐SY5Y cells. (A) Representative Western Blotting of immunoprecipitation (IP) showing the interaction between CREB and HDAC4 in SH‐SY5Y transiently transfected with EV or S1 Vector. IP with IgG was used as negative control. The input represents the pre‐immunoprecipitated total lysates (*n* = 3). (B, C) Gene and protein expression of CREB in SH‐SY5Y cells transiently transfected with S1 Vector for 48 and 72 h. *p ≤ 0.05 versus control (EV) by one‐way ANOVA analysis followed by Tukey's post hoc test (*n* = 3/4). (D) Western blotting images with quantification of CREB protein in SH‐SY5Y cells transiently transfected and treated with: (1) EV; (2) S1 Vector, (3) S1 Vector + siHDAC4, and (4) S1 Vector + MG132. *p ≤ 0.05 versus EV; ^#^ *p* ≤ 0.05 versus S1 Vector (48 h) by one‐way ANOVA analysis followed by Tukey's post hoc test (*n* = 3). (E) ChIP with CREB antibody followed by qPCR of the RIPK1 human promoter in SH‐SY5Y cells in the following conditions: (1) pGL3‐RIPK1, (2) pGL3‐RIPK1 + S1 Vector, (3) pGL3‐RIPK1 + S1 Vector + siHDAC4, and (4) pGL3‐RIPK1 + S1 Vector + MG132. *p ≤ 0.05 versus pGL3‐RIPK1; ^#^ *p* ≤ 0.05 versus pGL3‐RIPK1 + S1 Vector by one‐way ANOVA analysis followed by Tukey's post hoc test (*n* = 3). (F) Luciferase assay in SH‐SY5Y cells in the following experimental conditions: (1) pGL3basic, (2) pGL3‐RIPK1, (3) pGL3‐RIPK1 + S1 Vector, (4) pGL3‐RIPK1 + S1 Vector + siHDAC4, (5) pGL3‐RIPK1 + S1 Vector + MG132, (6) pGL3‐RIPK1 + S1 Vector + CREB Vector; *p ≤ 0.05 versus pGL3‐RIPK1; **p ≤ 0.05 versus all by one‐way ANOVA analysis followed by Tukey's post hoc test (*n* = 6). (G) Effect of siHDAC4 and CREB construct transfection or MG132 treatment on RIPK1 mRNA levels evaluated by qRT‐PCR in SH‐SY5Y cells after 48 h transfection of S1 Vector. ^#^ *p* ≤ 0.05 versus S1 Vector, ^##^ *p* ≤ 0.05 versus all by one‐way ANOVA analysis followed by Tukey's post hoc test (*n* = 3).

**FIGURE 5:**
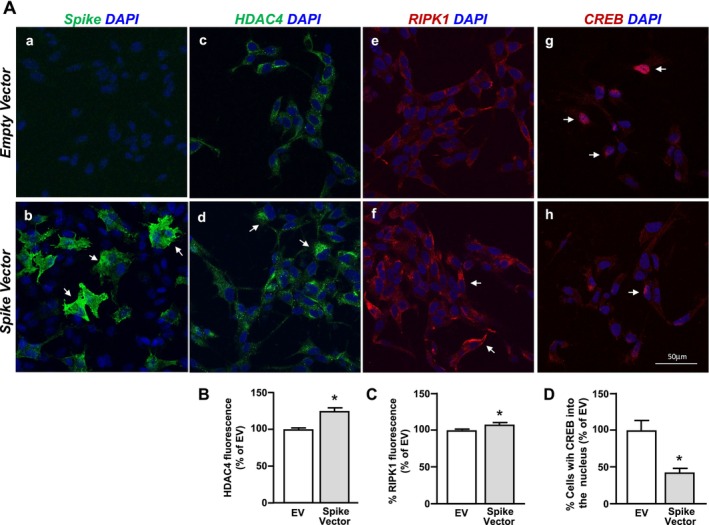
Effect of Spike S1 overexpression on HDAC4, RIPK2, and CREB expression in SH‐SY5Y cells. (A) (a, b) Representative confocal microscopic images displaying the distribution of S1 immunoreactivity in SH‐SY5Y transfected with empty vector (EV) (a) or S1 vector (b) for 72 h. (c–h) Representative confocal microscopic images displaying the distribution of HDAC4 (c, d), RIPK1 (e, f) and CREB (g, h) immunoreactivities in SH‐SY5Y transfected with EV (a) or S1 Vector (b) for 72 h. Arrows in (d, f) point to intense HDAC4 or RIPK1 immunoreactive cells; arrows in (g, h) point to cells with CREB+‐nucleus. Scale bars in (a–h): 50 μm. (B, C) Densitometric analysis of HDAC4+ (B) and RIPK1+ (C) fluorescence intensity in EV‐ or S1 Vector‐transfected SH‐SY5Y cells. (D) Quantitative analysis of cells with CREB+‐nucleus in EV‐ or S1 Vector‐transfected SH‐SY5Y cells. Data in (B–D) were expressed as percentage of EV. *p < 0.05 versus EV by unpaired Student's *t* test (*n* = 3).

Next, we investigated whether HDAC4 might bind to the promoter sequence of the RIPK1 gene. To this aim, SH‐SY5Y cells were co‐transfected with the Spike S1 Vector or the EV, and both with a plasmid containing the RIPK1 promoter sequence; 24 h after transfection, cell lysates were immunoprecipitated with IgG or HDAC4 antibodies. As shown in Figure 3A, the ChIP assay between the HDAC4 antibody and the RIPK1 promoter sequence showed no DNA binding, as compared to the negative control IgG. Since we previously found that RIPK1 is negatively regulated in a sequence‐specific manner by the transcriptional factor CREB in neurons [26], we investigated the putative interaction between HDAC4 and CREB after S1 Vector transfection. Interestingly, as revealed by the immunoprecipitation assay, S1 increased the binding between CREB and HDAC4 (Figure 4A). Intriguingly, we found a significant decrease of CREB at the protein level, but not at the transcriptional level, after 48 and 72 h of transfection with the S1 Vector (Figure 4B,C). It is known that HDAC4 can reduce the expression of transcription factors by causing their deacetylation and consequent ubiquitination [20]; to this aim, we evaluated the effect of siHDAC4 and the proteasomal inhibitor MG‐132 on CREB protein levels. Notably, both siHDAC4 and MG132 prevented the CREB protein reduction induced after 48 h of transfection with the S1 Vector (Figure 4D). To validate the functional effect of the CREB protein reduction induced by S1 Vector transfection—alone or after siHDAC4 and MG132 treatment—on the RIPK1 gene, ChIP experiments with the CREB antibody in cells co‐transfected with the S1 Vector and the plasmid containing the RIPK1 promoter sequence were performed. Significantly, siHDAC4 and MG132 counteracted the CREB binding reduction on the RIPK1 promoter sequence (Figure 4E). Remarkably, MG132 treatment and transfection of siHDAC4 or of the CREB Vector mitigated the effect of the S1 Vector to increase RIPK1 promoter activity and mRNA levels, as revealed by luciferase and qRT‐PCR experiments (Figure 4F,G). Notably, the CREB Vector significantly increased CREB expression, compared to the EV (Figure 3B). Consistently, confocal immunofluorescence analysis revealed that when SH‐SY5Y cells were transfected with the Spike vector (Figure 5A(a,b)), a significant increase in HDAC4 (Figure 5A(c,d),B) and RIPK1 immunofluorescence intensity was detected (Figure 5A(e,f),C), when compared to Empty Vector‐transfected cells. Conversely, the quantitative analysis revealed a significant reduction in the number of CREB^+^ nuclei in Spike Vector‐transfected cells (Figure 5A(g,h),D). These results suggest that Spike S1 overexpression, by increasing HDAC4, reduced CREB, which in turn up‐regulates RIPK1 at the transcriptional and translational levels.

### 3.4 S1 Spike Protein Is Internalized in the Cytoplasm via NRP1 Receptor Determining Neuronal Death in Differentiated SH‐SY5Y Cells

**FIGURE 6:**
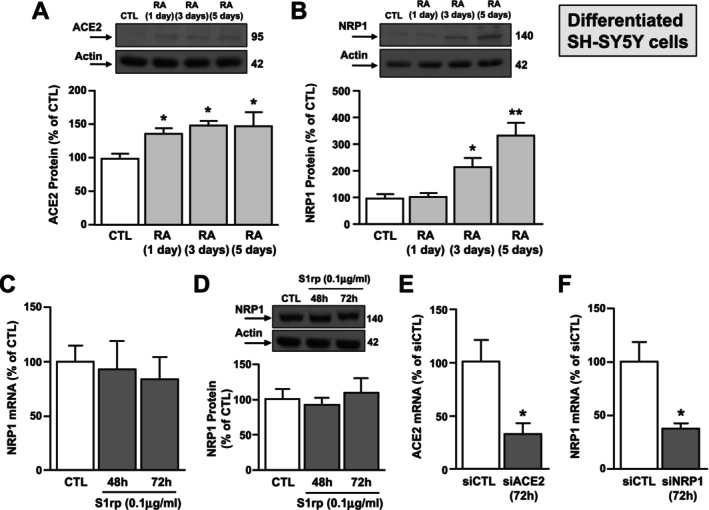
Effect of Retinoic Acid on ACE2 and NRP1 protein expression and of S1rp on NRP1 gene and protein expression in SH‐SY5Y cells. (A, B) Western blotting images with relative quantification of ACE2 and NRP1 protein levels in SH‐SY5Y cells differentiated with retinoic acid (RA) 10 μM for 1, 3 or 5 days. *p ≤ 0.05 versus CTL, **p ≤ 0.05 vs all by one‐way ANOVA analysis followed by Tukey's post hoc test (*n* = 3). (C, D) Effect of 48 and 72 h of S1rp treatment (0.1 μg/mL) on NRP1 gene and protein expression. *p ≤ 0.05 versus CTL by one‐way ANOVA analysis followed by Tukey's post hoc test (*n* = 3). (E, F) Effect of siRNAs against ACE2 (siACE2) and NRP1 (siNRP1) on ACE2 and NRP1 mRNA levels by qRT‐PCR in SH‐SY5Y cells differentiated with retinoic acid 10 μM for 5 days. *p ≤ 0.05 versus siCTL by Student's *t* test (*n* = 3).

**FIGURE 7:**
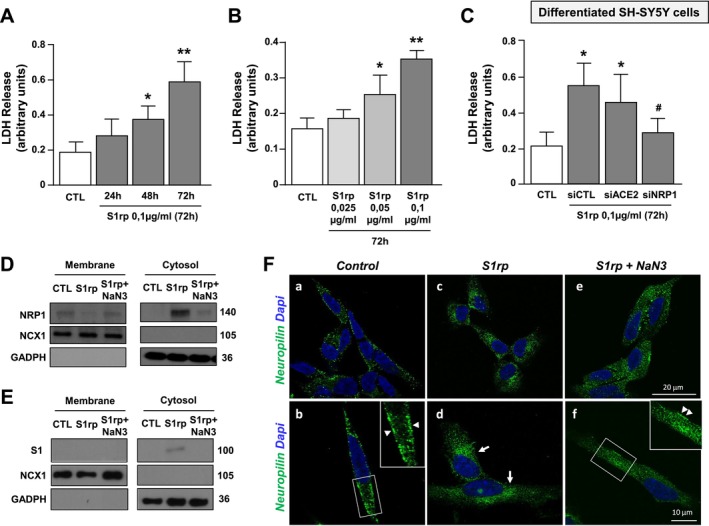
Effect of S1 recombinant protein (S1rp) to induce cell death via internalization of the NRP1 receptor in differentiated SH‐SY5Y cells. (A, B) LDH release assay in differentiated SH‐SY5Y cells treated with Spike‐S1rp: (A) at different times points (24, 48, and 72 h) at 100 ng/mL concentration and (B) at different concentrations (25–100 ng/mL) for 72 h. *p ≤ 0.05 versus Vehicle; **p ≤ 0.05 versus all by one‐way ANOVA analysis followed by Tukey's post hoc test (*n* = 4/5). (C) Effect of siACE2 and of siNRP1 on LDH release in cells exposed to 72 h of S1 protein (0.1 μg/mL). *p ≤ 0.05 versus Vehicle, ^#^ *p* ≤ 0.05 versus siCTL + Spike‐S1 protein (*n* = 5). (D, E) Western blot of NRP1 and Spike S1 subunit (S1) protein levels in membrane and cytosolic fraction of differentiated SH‐SY5Y exposed for 72 h to Spike‐S1 recombinat protein (100 ng/mL), alone or in combination with NaN_3_. GAPDH and NCX1 were used to verify the integrity of the cytosolic and membrane fraction of the samples (*n* = 3). (F) Representative confocal microscopic images displaying the distribution of neuropilin immunoreactivity in SH‐SY5Y cells under control conditions (a, b) and after exposure to Spike S1 recombinant protein, in the absence (c, d) or in the presence of NaN3 (e, f). Arrowheads in panels (b, f) point to neuropilin plasma membrane immunoreactivity. Arrows in (d) point to perinuclear neuropilin staining. Scale bars in (a, c, e): 20 μm; in (b, d, f): 10 μm.

To study the role of Spike S1 protein in a more specific neuron‐like experimental model, SH‐SY5Y cells have been differentiated with retinoic acid (RA, 10 μM) for 5 days and afterwards treated with 100 ng/mL of Spike S1rp [13]. Western Blot analysis revealed that ACE2 protein expression is very low and increased in differentiated SH‐SY5Y cells after 1, 3, and 5 days of treatment with RA. Remarkably, NRP1 is basally expressed in undifferentiated cells and its protein levels progressively increased after RA treatment (Figure 6A,B). Coherently, qRT‐PCR and Western Blot experiments demonstrated that NRP1 protein expression and mRNA levels are unmodified by S1rp treatment (Figure 6E,F). Furthermore, we studied the role of the ACE2 and NRP1 receptors in regulating S1rp‐induced LDH efflux. To this aim, cells were transfected with siRNAs against ACE2 (siACE2) and NRP1 (siNRP1) that were both able to significantly reduce their mRNA levels (Figure 6C,D). As shown in Figure 4A, a significant increase of LDH release occurred at 48 and 72 h of exposure, but not at 24 h (Figure 7A). Notably, dose–response experiments (0.025; 0.05 and 0.1 μg/mL) at 72 h demonstrated that cell death starts at 0.5 μg/mL and reaches the maximum effect at 100 ng/mL (Figure 7B) in neuron‐like SH‐SY5Y cells. Interestingly, siNRP1, but not siACE2, counteracted Spike S1‐induced cell death as revealed by LDH assay (Figure 7C).

Notably, cell fractionation experiments demonstrated that after S1rp treatment: (1) NRP1 protein expression was reduced at the plasma membrane level and increased into the cytoplasm, whereas (2) the S1 subunit was absent on the plasma membrane and appeared in the cytosol. Coherently, the pre‐treatment with the endocytosis inhibitor Sodium Azide (NaN3) strongly counteracted Spike S1 and NRP1 protein internalization in the cytoplasm (Figure 7D,E). In line, confocal immunofluorescence analysis showed that a punctuate NRP1 immunoreactivity was clearly detected along the plasma membrane of control SH‐SY5Y cells, while it was faintly observed within the cytosolic compartment. Conversely, a pronounced NRP1 immunosignal was observed within the cytosol, and not along the plasma membrane, in S1rp‐treated cultures. The pre‐treatment with NaN_3_ partially restored the plasma membrane NRP1 immunoreactivity (Figure 7F). These results demonstrated that Spike S1 protein via NRP1 internalizes in the cytosol thus causing cell death.

### 3.5 S1 Spike Protein and OGD/Rx Additively Increase the Neuronal Death via HDAC4/CREB/RIPK1 in Differentiated SH‐SY5Y Cells

**FIGURE 8:**
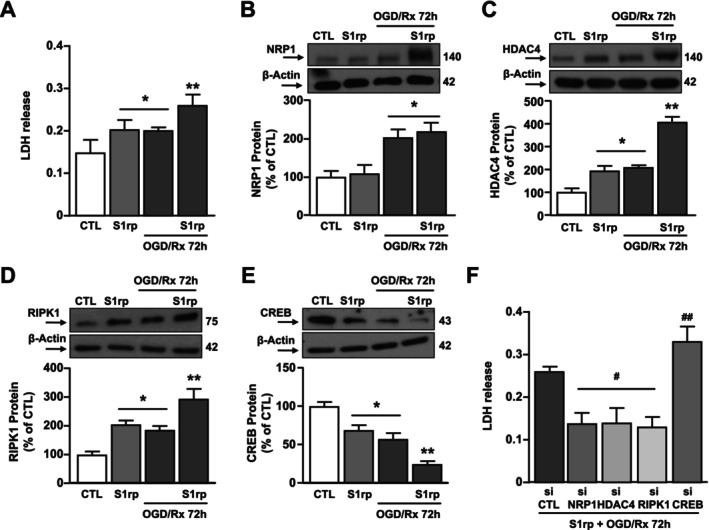
Effect of the knocking‐down of NRP1, HDAC4, RIPK1, and of CREB overexpression on neurodetrimental additive effect of S1 protein and oxygen and glucose deprivation (OGD) plus reoxygenation (Rx) in differentiated SH‐SY5Y cells. (A) LDH release measurement in differentiated SH‐SY5Y cells in the following conditions: (1) CTL; (2) treated for 72 h with S1rp (1 μg/mL); (3) subjected to 3 h of OGD and 72 h of Rx (OGD/Rx 72 h); (4) S1rp + OGD/Rx 72 h. *p ≤ 0.05 versus CTL; **p ≤ 0.05 versus all by one‐way ANOVA analysis followed by Tukey's post hoc test (*n* = 5). (B–E) Western blotting representative images and quantification of (B) NRP1, (C) HDAC4, (D) CREB, and (E) RIPK1 protein levels in cells in the above mentioned conditions. *p ≤ 0.05 versus CTL; **p ≤ 0.05 versus all by one‐way ANOVA analysis followed by Tukey's post hoc test (*n* = 5). (F) LDH release measurement in SH‐SY5Y cells subjected to OGD/Rx 72 h and treated with S1rp during the RX phase; 24 h before OGD/Rx procedures cells have been singly transfected with siCTL, siNRP1, siHDAC4, siRIPK1, siCREB. ^#^ *p* ≤ 0.05 versus S1rp + OGD/Rx72h + siCTL; ^##^ *p* ≤ 0.05 versus all by one‐way ANOVA analysis followed by Tukey's post hoc test (*n* = 5).

Since NRP1 [41], HDAC4 [21], CREB [42] and RIPK1 [43] protein dysregulation has been found to contribute independently to neuronal death in in vitro and in vivo stroke models [21] and that patients with COVID‐19 should undergo more aggressive stroke outcome [31, 44, 45], we examined whether S1rp and the experimental conditions mimicking brain ischemia OGD/Rx could have an additive effect in increasing neuronal death by modulating the NRP1/HDAC4/CREB/RIPK1 axis. To this aim, SH‐SY5Y cells were exposed to the following experimental conditions: (1) control, (2) S1rp (1 μg/mL) for 72 h, (3) OGD/Rx 72 h, (4) S1rp + OGD/Rx 72 h. LDH release experiments demonstrated an additive effect of S1rp and OGD/Rx treatment to induce neuronal death. Indeed, SH‐SY5Y subjected to both S1rp treatment and OGD/Rx showed an increase in extracellular LDH levels (74%) higher than either Spike (35%) or OGD/Rx alone (36%) (Figure 8A). Furthermore, Western Blot analysis showed that S1rp and OGD/Rx alone induced a significant: (1) increase of HDAC4 and RIPK1 and (2) reduction of CREB. Noteworthy, the combination of S1rp and OGD/Rx has an additive effect: (1) to upregulate HDAC4 and RIPK1 and (2) to downregulate CREB (Figure 8C–E). By contrast, NRP1 was not modified by single S1rp treatment but was up‐regulated by OGD/Rx alone or in combination with S1rp (Figure 8B). As shown in Figure 8F, the additive neurotoxic effects of S1rp and OGD/Rx treatments were abolished by siNRP1, siHDAC4, and siRIPK1, whereas they were significantly increased by CREB knocking down. These results confirm that the NRP1/HDAC4/CREB/RIPK1 biological mechanism is additively activated by S1rp and OGD/Rx to induce neuronal death.

## 4 Discussion

In this study, we tested the possibility that the mechanism by which SARS‐CoV2 induces neurotoxicity may be due to the direct effect of the Spike protein on neuronal cells; indeed, it has been detected in the post‐mortem brain of COVID‐19 patients [12, 46]. The viral glycoprotein Spike is composed of two subunits named S1 and S2; specifically, it has been demonstrated that S1 crosses the BBB by adsorptive transcytosis in a murine model [11]. Herein, Spike S1 vector transfection or S1rp exposures have been used to study possible effects of intracellular accumulation of the Spike S1 subunit in inducing neurotoxicity in SH‐SY5Y cells. Coherently, the same methods have been tested by other authors to confirm the intracellular role of Spike S1 in inducing cell toxicity. Indeed, it has been demonstrated that Spike: (1) promotes IL‐6 trans‐signaling by activation of the angiotensin II receptor in A543 epithelial cells [47]; (2) inhibits p53 activation in U2OS osteosarcoma cell line [48]; and (3) interacts with MAO‐B, impairing mitochondrial bioenergetics and inducing oxidative stress through mitophagy in SH‐SY5Y cells [49]. Notably, we found both that: (1) the transfection of the S1 construct in SH‐SY5Y cells and (2) the treatment with Spike S1rp induce necroptotic cell death by activating RIPK1. Notably, the up‐regulation of the necroptotic player RIPK1 mRNA and protein is the mechanism by which Spike‐S1 induced cell death. Indeed, both RIPK1 knocking down by siRNA transfection and its pharmacological inhibition via Nec‐1 cause a reduction of neurotoxicity. The correlation between the S1 subunit and necroptotic cell death has already been reported; in fact, S1 increases: (1) the phosphorylation of RIPK1 in differentiated adipocytes [50] and (2) the susceptibility of the 1‐methyl‐4‐phenyl‐1, 2, 3, 6‐tetrahydropyridine (MPTP) in inducing necroptosis in neuron‐like SH‐SY5Y cells [49].

Unraveling the role of the epigenetic erasers HDACs in S1‐induced RIPK1 up‐regulation, we found that only the HDAC4 isoform has a pivotal role. In fact, the knocking down of HDAC4 counteracts the S1‐dependent RIPK1 increase by mitigating the consequent neuronal death. HDAC4 can shuttle between the cytoplasm and nucleus [21], and its up‐regulation in both compartments has been demonstrated to be detrimental after neurotoxicant environmental pollutants exposure [19, 20, 28, 29]. Our results demonstrated that HDAC4 is not bound to the RIPK1 promoter sequence after S1 vector transfection, thus indicating a non‐direct regulation of RIPK1 by HDAC4. Indeed, HDAC4 increase occurs in the cytosolic compartment, where it interacts with the RIPK1 transcriptional repressor CREB [26], thus determining its reduction. Since it has been previously found that HDAC4 promotes ubiquitin‐dependent proteasomal degradation of the transcriptional factor Sp3 in SH‐SY5Y cells [20], we could speculate that the CREB decrease is a consequence of its HDAC4‐mediated deacetylation followed by ubiquitination. Indeed, S1‐induced reduction of CREB protein expression and of binding on the RIPK1 promoter were counteracted by the proteasome inhibitor MG132. Furthermore, overexpression of CREB caused a reduction in Spike S1‐induced RIPK1 up‐regulation and consequent neuronal death.

Notably, it has been demonstrated that Spike S1 neurotoxicity preferentially occurs via the activation of glia rather than a direct effect on neurons [51]. Noteworthy, we found that 72 h treatment with S1rp reduces cell survival in differentiated SH‐SY5Y cells, thus demonstrating Spike S1‐induced direct neuronal death. Regarding S1rp concentration (0.1 μg/mL) used in our experiments, it has already been tested: (1) in SH‐SY5Y cells causing a reduction of cell viability [52] and (2) mouse CLU199 immortalized hippocampal neurons [13]. Accordingly, other authors demonstrated by Multi‐Electrode Array (MEA) technique that S1rp, at the same range concentration used in our experiments, is responsible for reducing burst activities in populations of mouse cortical neurons [53]. Importantly, neuritic varicosities and reduction of burst activities are both markers of neuronal death; in fact, the first is a marker of neuronal damage in different neurodegenerative disorders, such as Alzheimer's disease (AD), Parkinson's disease (PD), amyotrophic lateral sclerosis (ALS) and traumatic brain injury [14]; the second has been found in cortical neurons treated with neurotoxic concentrations of glutamate [54].

Furthermore, cell fractionation and immunofluorescence experiments demonstrated that the S1 subunit was able to induce the translocation of the viral entry receptor NRP1 from the plasma membrane to the cytosol. Accordingly, Nrp1 is known to undergo clathrin‐dependent endocytosis in response to its ligand binding semaphorin 3A [55]. Previous studies showed that S1 crosses the BBB and that ACE2 and NRP1 are involved in the binding and internalization of SARS‐CoV2 into the host cells [7].

Herein we assessed that NRP1 is an important player involved in Spike S1‐induced neurotoxicity; indeed, NRP1 knockdown, but not ACE2, prevents the HDAC4/CREB/RIPK1 axis activation mediated by Spike S1. Accordingly, it has been found that NRP1 inhibitor, but not recombinant soluble ACE2, protects against neurite shortening in neuronal‐like SH‐SY5Y cells [56]. Notably, Spike S1 has an effect on modulating NRP1 mRNA and protein levels, but decreases NRP1 on the cell membrane, thus increasing its presence in the cytosol. Coherently, the endocytosis inhibitor NaN3, by blocking Spike S1‐induced NRP1 internalization, determines a strong expression of NRP1 at the cell membrane surface, as revealed by cell fractionation and immunofluorescence experiments.

The lack of the effect of siACE2 in modulating Spike S1‐induced neurotoxicity could be related to its protein levels; indeed, we found that ACE2 is expressed at very low levels and is slightly increased after RA treatment. This result is in accordance with The Human Protein Atlas (https://www.proteinatlas.org) showing that ACE2 RNA has a little expression in the CNS and is majorly present in the heart and kidney [57]. Importantly, other authors demonstrated that ACE2 protein is not detected [58] or is faintly expressed in SH‐SY5Y cells [59].

On the other hand, it has already been demonstrated that there is a significant worsening outcome of acute ischemic stroke in COVID‐19 patients, due to different mechanisms involving a prothrombotic state, changes in lipid metabolism and platelet aggregation, alteration in endothelial function, and plaque instability and rupture [45]. This study reveals for the first time that Spike S1 and OGD/Rx show an additive effect in inducing necroptosis in a neuronal cell line. Both neurotoxic stimuli exert their effect through the activation of the same biochemical pathway. Indeed, we found that OGD/Rx and Spike S1 independently activated the NRP1/HDAC4/CREB/RIPK1 axis and that the combination of both neurotoxic stimuli additively increased neuronal death. Indeed, single siRNA transfection of each component of the NRP1/HDAC4/CREB/RIPK1 axis mitigates the cell death induced by the combination of S1rp and OGD/Rx treatments. This paper provides new insights into the molecular mechanisms through which S1‐induced neurotoxicity could be an additional risk factor worsening the stroke‐associated neurodegeneration.

## Author Contributions

All authors contributed to the study conception and design. Material preparation, data collection, and analysis were performed by Luca Sanguigno, Natascia Guida, Mariarosaria Cammarota, Silvia Ruggiero, Angelo Serani, Francesca Galasso, Vincenzo Pizzorusso, and Francesca Boscia. The first draft of the manuscript was written by Luigi Formisano, Luca Sanguigno, Natascia Guida, Silvia Ruggiero, and all authors commented on previous versions of the manuscript. All authors read and approved the final manuscript.

## Conflicts of Interest

The authors declare no conflicts of interest.

## Data Availability

Data will be made available on request.
